# The Potential of Wild Yeasts as Promising Biocontrol Agents against Pine Canker Diseases

**DOI:** 10.3390/jof9080840

**Published:** 2023-08-11

**Authors:** Eugenia Iturritxa, Nebai Mesanza, María-Jesús Torija

**Affiliations:** 1 Department of Forest Science, Neiker-BRTA, Instituto Vasco de Investigación y Desarrollo Agrario, Granja Modelo s/n, Antigua Carretera Nacional 1, Km. 355, 01192 Arkaute, Spain; nmesanza@neiker.eus; 2 Grup Biotecnologia Enològica, Departament de Bioquímica i Biotecnologia, Facultat d’Enologia, Universitat Rovira i Virgili, c/Marcel⋅lí Domingo 1, 43007 Tarragona, Spain

**Keywords:** wild yeast, pitch canker, Diplodia shoot blight, biological control, tolerant clones

## Abstract

Native wild yeasts from forest ecosystems represent an interesting potential source of biocontrol organisms in synergy with disease-tolerant forest materials. Yeasts have a combination of competitive mechanisms and low requirements for their biotechnological application as biocontrol agents. The current study aimed to increase the number of biocontrol candidates against *Fusarium circinatum* and *Diplodia sapinea*. The enzymatic and antagonistic activities of the biocontrol candidates were evaluated using different screening methods, in which the direct impact on the growth of the pathogen was measured as well as some properties such as cellulose and lignin degradation, tolerance to biocides, volatile compound production, or iron effect, which may be of interest in biotechnological processes related to the management of forest diseases. A total of 58 yeast strains belonging to 21 different species were obtained from oak forest and vineyard ecosystems and evaluated. The application of yeast treatment behaved differently depending on the pathogen and the plant clone. The 2g isolate (*Torulaspora delbrueckii*) showed the highest inhibitory activity for *D. sapinea* and 25q and 90q (*Saccharomyces paradoxus*) for *F. circinatum*. Clones IN416 and IN216 were the most susceptible and the most tolerant to *D. sapinea*, respectively, while the opposite was observed for *F. circinatum*.

## 1. Introduction

The modern applications of yeasts have expanded considerably beyond the traditional uses in the food fermentation process; yeasts are utilized in agriculture and other fields such as environmental applications, bioremediation, compost improvement, biocontrol agents, and even medical and pharmaceutical uses due to the heterogeneous production of enzymes and pharmaceutical proteins. Considering the genetic, phenotypic, and metabolic diversity of yeast species, there is a vast potential yet to be discovered for the production of worthy products and applications [1]. Specifically, there is increasing attention on the biotechnological applications of yeast as biocontrol agents, as they have been reported to be potent antagonists of various plant pathogens through their competitive mechanisms involving enzyme secretion, the production of secondary metabolites or toxins, and the induction of resistance. In addition, their low culture requirements and fast multiplication make them good candidates for biocontrol and other biotechnological applications [1].

The application of chemicals to reduce forest disease impact is no longer an option. The European Community establishes rules for the sustainable use of agrichemicals to reduce the risks and impacts on human and environmental health through EU Directive 2009/128/CE [2]. In June 2022, as part of the Green Deal, the European Commission adopted a proposal to restore damaged ecosystems and nature in Europe’s farmlands, seas, forests, and urban environments by 2050. As part of this, the Commission proposes reducing chemical pesticides usage by 50% by 2030. In this context, pest control with biocontrol agents has high potential and alternatives, which can be incorporated into sustainable pest control practices in agronomy and forestry [3,4].

Forest diseases are spreading as a result of human interventions, such as monospecific plantations, the use of a reduced number of highly productive forest species, the increase in global international trade [5], and climate change, which promotes the development of diseases and vectors in unexpected zones [6,7]. Among them, pitch canker, caused by an introduced species, *Fusarium circinatum* [8], and Diplodia shoot blight, caused by *Diplodia sapinea* [9], are two of the most important pine diseases. They are responsible for major losses in regions that depend on a monospecific pine economy [10,11].

*Fusarium circinatum* causes cankers with noticeable resin exudates that girdle branches and trunks of *Pinus* species. Multiple-branch infections may cause severe crown dieback and even root infections [12] and eventually, the death of infected trees. It has been found in regions of America, Asia, Africa, and Europe. *D. sapinea* also causes a range of disease symptoms in coniferous trees, including crown wilt, bark cankers, root diseases, and damping-off and root rot of seedlings. In addition, this species causes the blue stain of sapwood on fallen or cut timber. This fungus has a wide distribution in temperate and tropical regions [13]. Mycoviruses are considered potential biocontrol agents against *D. sapinea*. In fact, two dsRNA mycoviruses have been identified in this species, RNA virus 1 and 2 (SsRV1, SsRV2, respectively; Totiviridae) [14]. In the case of *F. circinatum*, three putative *Mitovirus* spp. (Narnaviridae) were identified, named *F. circinatum* mitovirus 1, 2-1, and 2-2 (FcMV1, FcMV2-1, and FcMV2-2) [15]. Some bacteria (*Pseudomonas fluorescens*, *Bacillus simplex,* and *Erwinia billingiae*) and essential oils from *Cinnamomum verum* and *Syzygium aromaticum* have shown promise as prophylactic treatments to reduce the devastating effects of *F. circinatum* and *D. sapinea* on *Pinus radiata* [16]. The fungal species most used in biocontrol are *Trichoderma virens* Mater., *T. viride* Pers., and *T. harzianum* Rifai, with the latter being the most commercially used and the most investigated for its application in biological control [17,18], and it has already been tested against *F. circinatum* and *D. sapinea* [19,20]. However, the negative effects of these *Trichoderma* species on plants have been observed, as they cause a decrease in the germination rate of seeds, also in *P. radiata* [21,22].

A crucial step in the development of products based on biological control agents is the identification and selection of suitable candidates by evaluating their biological properties. Included in the biological properties of interest are nutrient or space competition [23], iron depletion [24], extracellular lytic enzymes production [25], and volatile organic compounds [26], among others. Inhibition capabilities on mycelial growth or conidia germination in molds have been reported for some yeast strains [27]. Endophyte-derived volatile organic compounds are active in disease control as direct antimicrobial agents and resistance inducers, preventing plant colonization by pathogens [28].

The current study aimed to increase the number of biocontrol candidates against *F. circinatum* and *D. sapinea* using novel approaches for the selection of natural epiphytic yeasts. In addition, to increase biological control success, host tolerance was also evaluated to integrate sustainable management strategies. The antagonistic activities of the biocontrol candidates were evaluated using different screening methods, in which the direct impact on the growth of the pathogen was measured as well as some properties, such as cellulose and lignin degradation, tolerance to biocides, volatile production, or the iron effect, which may be of interest in biotechnological processes related to the management of forest diseases. Furthermore, the biocontrol efficiency of the selected candidates on their target organisms under in vitro and in vivo conditions, as well as their impact on the host and the surrounding flora, was evaluated.

## 2. Materials and Methods

### 2.1. Microorganisms and Growth Conditions

The 58 yeast strains evaluated in this study were isolated from grapes and oak forests in the Basque Country, Spain, during 2019 (Table 1) and were identified and characterized in a previous study [29]. These yeasts were stored in 2 mL cryovials containing 25 porous beads and cryopreservative broth (Deltalab, Spain) at −20 °C until use. Fresh yeast cultures were obtained by inoculating 1% (*v*/*v*) of the cryopreservative broth stocks on yeast peptone dextrose (YPD) (Condalab, Madrid, Spain) agar and YPD broth (Condalab) (25 mL in sterile 50-mL conical tubes) at 25 °C for 3 days in an orbital shaker (S100D Optic Ivymen System, Barcelona, Spain) at 200 rpm. Then, yeast cells were harvested by centrifugation at 3000 rpm (Centrifuge 4k15, SIGMA, Germany) for 5 min at 4 °C and washed twice with sterile water. For the different tests, the yeast suspensions were adjusted to 10^6^ cells/mL [30]. Erythrosine dye was used to detect cell viability. Briefly, 1 g of erythrosine was dissolved in 100 mL of distilled water; then, 1 mL of this solution was added to a final volume of 50 mL of a buffer solution prepared with 0.2 M Na_2_HPO_4_ and 0.2 M NaH_2_PO_4_ (1:1) to obtain a 1:5000 dye solution [31]. The viable cells were counted using a Neubauer chamber, under a NIKON DS-Fi1 microscope with a 40X objective.

In this work, two fungal strains were used to perform antagonistic studies: a strain of *F. circinatum*, CECT20759, (CECT, Colección Española de Cultivos Tipo, Burjassot, Valencia, Spain), and a strain of *D. sapinea*, DPV24, (Neiker collection). These fungi were selected based on their virulence determined by artificial inoculation on pine seedlings and for being the representative haplotypes of the pathogen populations in the Basque Country (Spain). On the one hand, the *F. circinatum* strain was isolated from a *P. radiata* plantation in Gipuzkoa (Spain) and was associated with vegetative compatibility group (VCG) A and mating type 2 (Mat-2), the only mating type and haplotype present in the Basque Country [32]. On the other hand, the *D. sapinea* strain was isolated from an adult *P. radiata* tree from Bizkaia (Spain) [33]. Fungal cultures were plated on potato dextrose agar medium (PDA) (Condalab), and incubated at 20 °C in darkness for 7 days.

### 2.2. Estimation of Ligninolytic and Cellulolytic Enzyme Activities

Ligninolytic enzymes are the key enzymes for the degradation of lignin. Three different media were utilized to assess the ability of the isolates to degrade lignin:

(i) Lignin medium I. Sodium lignosulfonate agar medium: 0.2% sodium lignosulfonate, 2 g of (NH_4_)_2_SO_4_, 1 g of K_2_HPO_4_, 1 g of KH_2_PO_4_, 0.2 g of MgSO_4_, 0.1 g of CaCl_2_, 0.05 g of FeSO_4_, 0.02 g of MnSO_4_, 20 g of agar, and 1 L of tap water (pH = 7.0).
(ii) Lignin medium II. Aniline blue agar medium: 10 g of yeast extract, 20 g of glucose, 20 g of agar, 0.1 g of aniline blue, and 1 L of distilled water.
(iii) Lignin medium III. Remazol brilliant blue agar medium: 10 g of yeast extract, 20 g of glucose, 20 g of agar, 0.1 g of Remazol brilliant blue, and 1 L of distilled water [34,35].

The screening for lignin-degrading yeast was carried out in two steps. Yeasts were first inoculated onto lignin medium I using the streak plate method and incubated at 28 °C for 5 days. Potential lignin-degrading yeasts were initially detected according to colony growth. We used lignosulfonate in this medium, because its chemical structure is almost the same as that of natural lignin. Yeasts were then transferred to lignin media II and III and incubated at 28 °C for 4 days. The ligninolytic enzyme activity of the isolates was measured based on the fact that the higher the laccase (Lac), manganese peroxidase (MnP), and lignin peroxidase (LgP) activity of the studied isolates, the stronger their ability to degrade lignin. Aniline blue discoloration (lignin medium II) is related to the activity of LgP and MnP enzymes, while the Remazol brilliant blue discoloration (Lignin medium III) is related to Lac enzyme activity. Therefore, the lignin degradation ability of yeast isolates was directly proportional to the size of the discolored area; the larger the discolored area, the higher the lignin degradation capacity of yeast isolates [34]. Three replicates were performed for each isolate.

The cellulose degradation capacity of the yeast isolates was tested on sodium carboxymethyl cellulose (CMC-Na) agar plates (15 g of CMC-Na, 5 g of NaCl, 1 g of KH_2_PO_4_, 0.2 of g MgSO_4_, 10 g of peptone, 5 g of yeast extract, 18 g of agar, and 1 L of distilled water). Yeast isolates were cultured on this medium in triplicate and incubated at 28 °C for 4 days. After incubation, plates were flooded with 1 g/L of Congo red for 15–20 min. Then, the dye was discarded, and plates were flooded with 1 M NaCl for 20 min. Finally, the NaCl solution was discarded [36,37]. The Congo red dye binds to the polysaccharide substrate in the medium and therefore, a halo zone around the colony indicates cellulolytic enzyme activity of the isolate. The activity was measured based on the size of this halo; the larger the halo, the greater the cellulose degradation capacity of the isolate.

### 2.3. Determination of Biocide Effectiveness by Inhibition Assay

The tolerance of the 58 yeast isolates to biocides was tested. Eight antimicrobial agents were included in a multidisc system. Yeast Multidisc 95280 (Liofilchem; Italy): caspofungin (5 µg), fluconazole (25 µg), posaconazole (5 µg), voriconazole (1 µg), amphotericin B (20 µg), ketoconazole (10 µg), flucytosine (1 µg), and nystatin (100 µg). The combination of these chemicals is considered a broad-range biocide for yeast. Plates were examined for halo zones around the isolate colony after incubation at 28 °C for 3 days. The diameter of the halos indicated the effectiveness of the biocidal agent and was classified into 4 categories depending on the size: Code (1) 2 mm; (2) >2–4 mm; (3) >4–6 mm; (4) >6 mm. Each antimicrobial agent test comprised three replicates.

### 2.4. Yeast Production of Hydrogen Sulfide and Acetic Acid

The production of hydrogen sulfide and acetic acid was determined in the 58 yeast isolates. First, 100 µL of yeast cell suspensions (10^6^ cells/mL) were spotted on Biggy Agar (Oxoid, Bakingstoke, UK) and on CaCO_3_ agar medium (5 g/L yeast extract; 20 g/L glucose; 10 g/L CaCO_3_; 20 g/L agar) to determine H_2_S and acetic acid production, respectively. The plates were incubated at 30 °C for 3 days. The qualitative amount of H_2_S production was determined by the color of the colonies, ranging from white (no release) to black (high release). The codification used was as follows: (0) white (undetected), (1) light brown, (2) brown, (3) dark brown, and (4) black [38].

In the case of acetic acid, a halo around colonies growing on CaCO_3_ agar medium means that the yeast isolates produce acetic acid. The isolates were classified according to the diameter of the halo: (0) no acetic acid detected; (1) ≤1 mm; (2) >1 mm and ≤2 mm [38]. Both tests were implemented in three replicates.

### 2.5. In Vitro Assays for Antagonistic Activity

To evaluate the antagonistic capacity of the 58 yeast isolates against the selected strains of *F. circinatum* and *D. sapinea*, the technique of paired cultivation in Petri dishes was used [39]. For this purpose, 2-day-old yeast cultures were plated as streak lines on YPD agar plates with 25 mm between them. A mycelial disc (5 mm in diameter) of an actively growing culture of the tested pathogenic fungus was placed in the center at a standard distance and incubated at 25 °C for 7 days in darkness (Incubator Sanyo MLR-35H, Japan). Controls consisted of plates inoculated with *F. circinatum* and *D. sapinea,* but without the yeast.

To evaluate the inhibition, the average diameters of the *F. circinatum* and *D. sapinea* colonies were measured using ImageJ software. A completely randomized design with three replicates was used for each of the trials. Yeast isolates that showed inhibitory activity against pathogen growth were chosen for further testing, such as analyzing the effect of iron concentrations on the inhibitory activity of yeast isolates and in vivo assays of plant pathogenicity and antagonism.

### 2.6. Effect of Iron Concentration on Yeast Inhibitory Activity

The effect of iron concentration on the antagonistic activity of yeast isolates was evaluated by measuring differences in pathogen growth in the presence or absence of iron [24]. First, yeasts were cultured on PDA plates with (5 and 20 μg/mL) and without FeCl_3_, using the paired cultivation technique explained in the previous section. Plates were incubated at 25 °C for 7 days. Controls consisted of plates inoculated with *F. circinatum* and *D. sapinea* without the yeast. A completely randomized design with three replicates was used for each of the trials. The average diameters of the *F. circinatum* and *D. sapinea* colonies were measured using the ImageJ software.

### 2.7. Phytopathogenicity Test on Plants

To define the potential pathogenicity of wild yeast isolates on plants, Spirodela duckweed toxicity test and Phytotoxkit liquid samples (Ecotest, Valencia, Spain) were used, following the manufacturer’s instructions. These cost-effective and user-friendly phytotoxicity assays strictly adhered to ISO 20227 and 18763 Standards, respectively.

The Spirodela duckweed toxicity test was used to assess growth inhibition and photosynthetic pigment reduction in the “first frond” (the largest frond of each turion) of *Spirodela polyrhiza* after a 3-day exposure to a yeast suspension. Growth was determined by measuring the area of the first frond, at T = 0 and T = 72 h, with the aid of image analysis.

The Phytotoxkit liquid samples were used to perform a 3-day seed germination and early growth with the monocot *Sorghum saccharatum* (Sorgho) and the dicots *Lepidium sativum* (garden cress) and *Sinapis alba* (mustard) in three replicates (10 seeds per replicate). In this case, 20 mL of the yeast suspension was spread over the entire surface of the thick, white filter paper, following the manufacturer’s instructions. The three species are frequently used in phytotoxicity assays and were selected for the Phytotoxkit microbiotest due to their rapid germination and root and shoot growth, allowing observations and scoring after only 3 days.

Both experiments allow us to understand the potential negative impact of these microorganisms on the adjacent or coexisting flora in plantations or nurseries, in which yeast could be used as biocontrol agents.

### 2.8. In Vivo Assays for Antagonistic Activity

The five yeast isolates that showed evident inhibitory activity in in vitro assays (2g, 25q, 49q, 58q, and 90q) were selected for the in vivo test. Biocontrol tests of *D. sapinea* and *F. circinatum* isolates were assessed using potted cuttings (four clones) of *P. radiata*. Healthy cuttings of *P. radiata* (from Basque Country) growing in a mixture of 60% clay, 20% sand, and 20% humus were placed in a P2 biosecurity greenhouse at 20 °C under 16 h of light for 28 days prior to inoculation. They were irrigated with tap water at 48 h intervals. Four replicates per treatment and clone were performed. Cuttings were inoculated into the wound made on the stem of each cutting after using a sterilized scalpel to remove the shoot tips. Wounds were previously, 24 h before, inoculated with yeast cell suspensions (adjusted to a population of 6 × 10^6^ cells/mL) [30], and then equal-sized agar plugs colonized by *D. sapinea* or *F. circinatum* were inserted and wrapped with Parafilm. Cuttings inoculated with only yeast cells, only pathogens, or with noncolonized agar plugs were used as controls. Additional controls of only water were used to evaluate the impact of mechanical wound damage on plants [40]. A randomized complete block experimental design was used in the trial. Inoculated cuttings were incubated in a P2 biosecurity greenhouse for 4 weeks, after which the bark was removed with a scalpel and the disease severity was evaluated by measuring the lesions with an electronic caliper [16].

### 2.9. Statistical Analysis

Data were analyzed using SPSS18.0 (SPSS Inc., Chicago, IL, USA) and XLStat 2022.3.2 (Addinsoft 2022) statistical packages. The data were subjected to experimental design and statistical analysis. Descriptive data are presented as the mean ± standard deviation (mean ± SD).

Data on the estimation of ligninolytic and cellulolytic enzyme activities, biocide effectiveness, and hydrogen sulfide and acetic acid production were subjected to Hierarchical clustering using the unweighted pair group method with arithmetic mean (UPGMA) algorithm, applying the Euclidean distance measure to transform the similarity coefficients into distances, and producing dendrograms [41]. Data from in vitro and in vivo assays for antagonistic activity, iron inhibitory activity, and pathogenicity were subjected to a general linear model and univariate or multivariate analysis of variance (ANOVA and MANOVA). Intergroup differences were considered statistically significant at *p* ≤ 0.05 and highly significant at *p* ≤ 0.001. Significant differences between means were determined using the Tukey HSD test with a significance level of 0.05 [42].

## 3. Results

### 3.1. Determination of Ligninolytic and Cellulolytic Enzyme Activities

**Figure 1:**
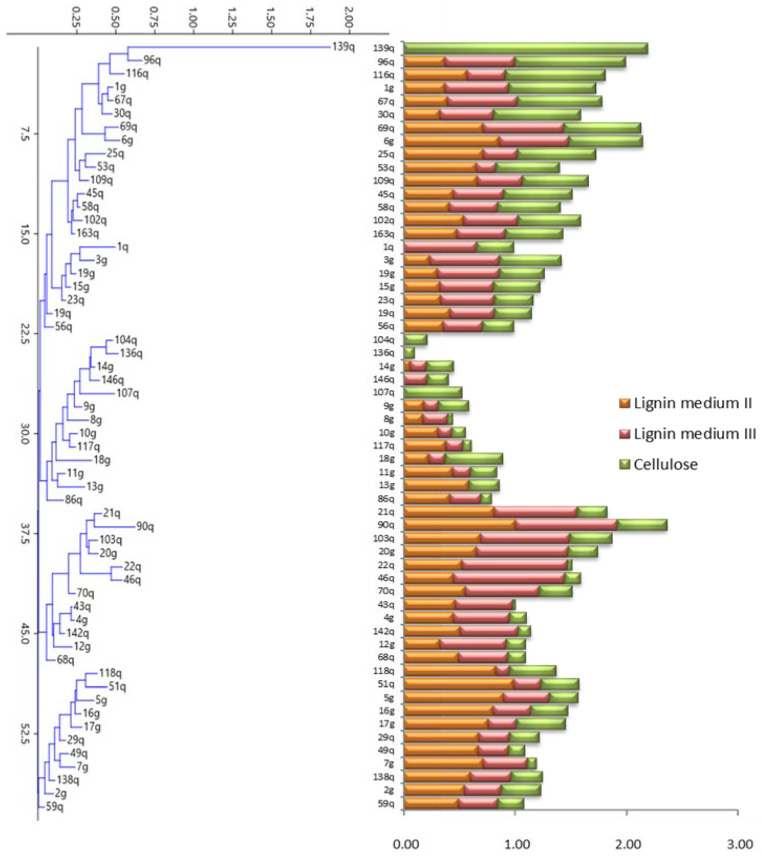
Dendrogram and bar plot representing the clustering of yeast isolates based on their ligninolytic and cellulolytic activities (Lignin medium II = represents the Lgp and Mnp activities present in the isolates and Lignin medium III = represents the Lac activity of the isolates).

All yeast strains were grown in a medium containing sodium lignosulfonate (Lignin medium I) (data not shown). These strains were then transferred to a medium with aniline (Lignin medium II) and Remazol (Lignin medium III). In both media, the strains 104q (*Lachancea thermotolerans*), 107q (*Pichia kudriavzevii*), 136q, and 139q (both belonging to the *Pichia manshurica* species) did not show discolored areas, indicating a lack of LgP, MnP, or Lac activities. On the other hand, strain 146q (*P. kudriavzevii*) showed weak Lac activity (1.99 mm) and no LgP and MnP activities, while strain 13g (*L. thermotolerans*) was among the strains with higher LgP and MnP activity (5.73 mm) but without Lac activity. The rest of the strains presented different sizes of discolored areas, indicating that they had activity in the three enzymes (LgP, MnP, and Lac), although at different intensities. Among the strains that showed the highest lignin degradation capacity (>8.00 mm), 118q, 51q (both *L. thermotolerans* species), 6g (*Hyphopichia pseudoburtoni*), and 5g (*Naganishia albida*) were the most active strains for LgP and MnP activities, and 20g (*Saccharomyces cerevisiae*), 22q (*Torulaspora delbrueckii*), and 46q (*Saccharomyces paradoxus*) were the more active strains for Lac activity. Finally, strain 90q (*S. paradoxus*) showed a strong response in both media, indicating a high activity of the three enzymes (Figure 1, Supplementary Table S1, Supplementary Figure S1a).

All isolates were cultured on a medium containing CMC to detect to what extent they could degrade cellulose (Figure 1, Supplementary Table S1, Supplementary Figure S1b). Some isolates barely grew on this medium and presented a reduced degradation halo, but at least a weak signal was detected in all the strains (halos of approximately 2 mm). The strains with the highest activity (>7 mm) were 116q (*Meyerozyma caribbica*), 96q (*S. paradoxus*), and 139q (*P. manshurica*), and the last strain stood out with an activity (14.39 mm) that doubled that of those considered highly active.

With all these data, a dendrogram was made (Figure 1), showing that the isolates were mainly distributed according to their cellulolytic activity since those with high cellulolytic activity were placed in the upper part of the dendrogram, while those with low cellulolytic activity were located in the lower part of the dendrogram. Finally, the isolates with low activity in all the tested enzymes were located in the central part of the dendrogram.

### 3.2. Determination of Biocide Effectiveness by the Inhibition Assay

**Figure 2:**
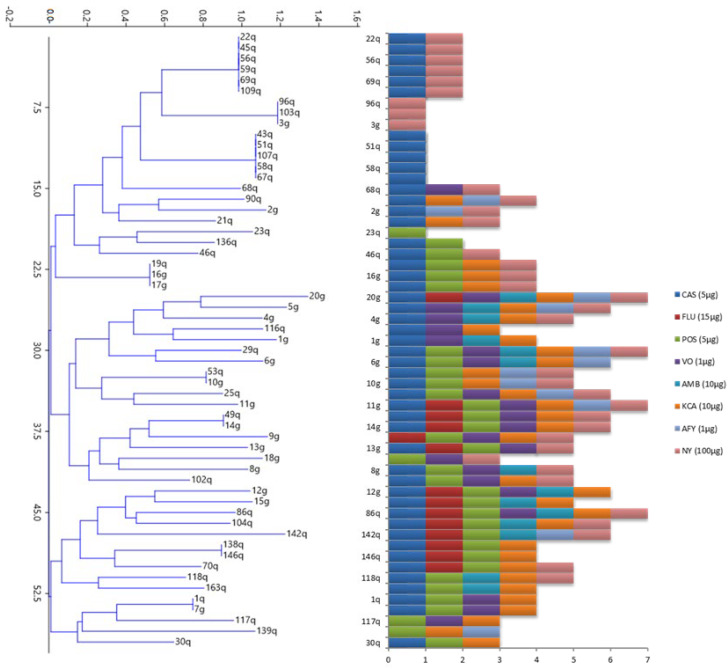
Dendrogram and bar plot representing the clustering of yeast isolates based on their biocide susceptibility to the different tested substances: fluconazole (FLU), posaconazole (POS), voriconazole (VO), amphotericin B (AMB), ketoconazole (KCA), flucytosine (AFY), and nystatin (NY).

All the isolates were sensitive to at least one biocide, mostly to caspofungin (>86% of the isolates), nystatin (65%), and ketoconazole and posaconazole (60% of the isolates). In the case of fluconazole, amphotericin B, and flucytosine, less than 25% of the strains showed susceptibility (Figure 2, Supplementary Table S1, Supplementary Figure S1c). However, the intensity of the effect produced by the different agents on this growth suppression was different, and the efficacy of the individual and combined agents varied between the tested strains.

Among the strains with the highest tolerance to the tested biocides, 51q, 103q (both *L. thermotolerans* species), 96q (*S. paradoxus*), and 3g (*T. delbrueckii*) stood out. In contrast, strains 20g (*S. cerevisiae*), 29q, 86q (*S. paradoxus*), and 11g (*Hanseniaspora uvarum*) showed the greatest sensitivity to the tested biocides. 

The dendrogram showed the different clusters of yeast that were formed based on their biocide activity, locating the most susceptible strains to the tested biocides in the middle area and the most resistant ones in the upper part of the dendrogram (Figure 2).

### 3.3. Production of Acetic Acid and Hydrogen Sulfide

**Figure 3:**
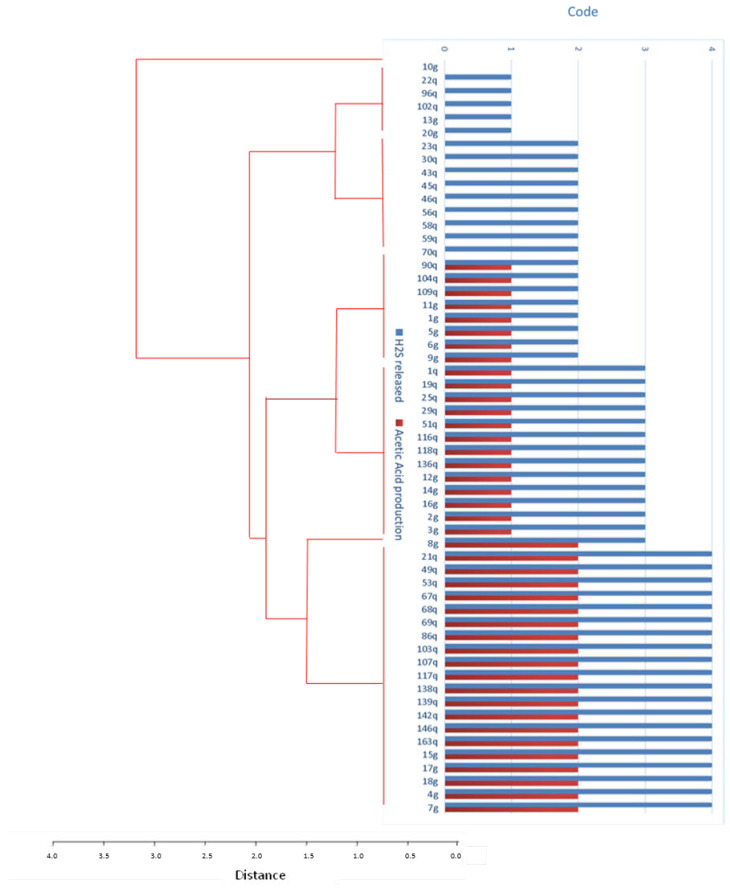
Dendrogram and bar plot representing the clustering of yeast isolates based on their production of acetic acid and hydrogen sulfide.

All the isolates produced hydrogen sulfide, except the 10g isolate (*Starmerella bacillaris*), which did not produce this compound or acetic acid. Indeed, in 26% of the isolates, acetic acid was not detected, and when it was detected, the amount was generally low, with a maximum of two on a scale up to four (Figure 3, Supplementary Table S1, Supplementary Figure S1d,e). Seven yeast clusters were formed by combining the production of acetic acid and hydrogen sulfide, from the total absence of synthesis of both compounds, only observed in 10g, to the detection of high production in both cases (especially for hydrogen sulfide, with a four on a four scale) in 35.1% of the studied yeast isolates.

### 3.4. In Vitro Assays for Antagonistic Activity

**Figure 4:**
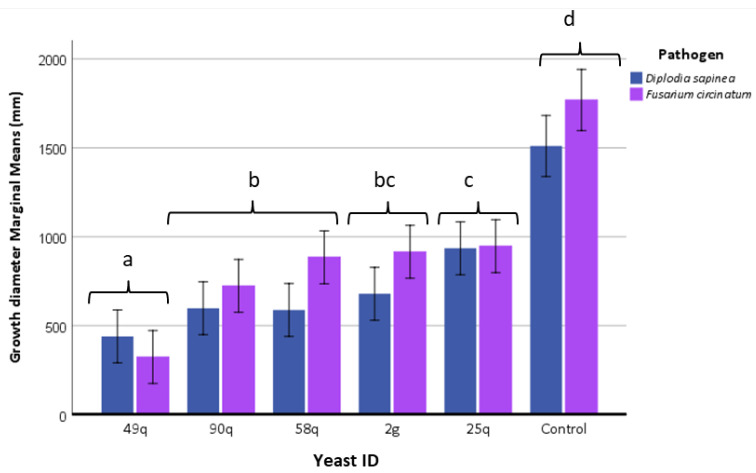
Growth (colony diameter measurement) of pathogens *Diplodia sapinea* and *Fusarium circinatum* using the technique of paired cultivation in the presence of five yeast isolates. The growth of these pathogens in the absence of yeast was used as a control. Statistically significant differences (*p* < 0.05) among treatments are indicated with different lowercase letters. Bars indicate error, 95% confidence intervals.

All yeast isolates were included in an in vitro assay to evaluate their antagonistic capacity against *F. circinatum* and *D. sapinea* (Supplementary Figure S2). Five of the fifty-eight yeast isolates showed an inhibitory effect on the growth of the two tested pathogenic fungi, 2g (*T. delbrueckii*) isolated from *Vitis vinifera* and 25q, 49q, 90q (all *S. paradoxus* species), and 58q (*Kluyveromyces dobzhanskii*) from native oak ecosystems (Figure 4). The 49q isolate showed the greatest effectiveness under in vitro conditions by reducing the growth of the pathogens by more than 75% compared to the control (pathogen grown in the absence of yeast). Nevertheless, the reduction in the growth of the pathogenic strains produced by 90q, 58q, 2g, and 25q yeasts was also notable, approximately 50% in most cases. Statistically significant differences were detected in the growth diameter of the pathogenic strains among yeast isolates (*p* < 0.001), between pathogenic strains (*p* = 0.05), and from the interaction between yeasts and pathogens (*p* < 0.05).

**Figure 5:**
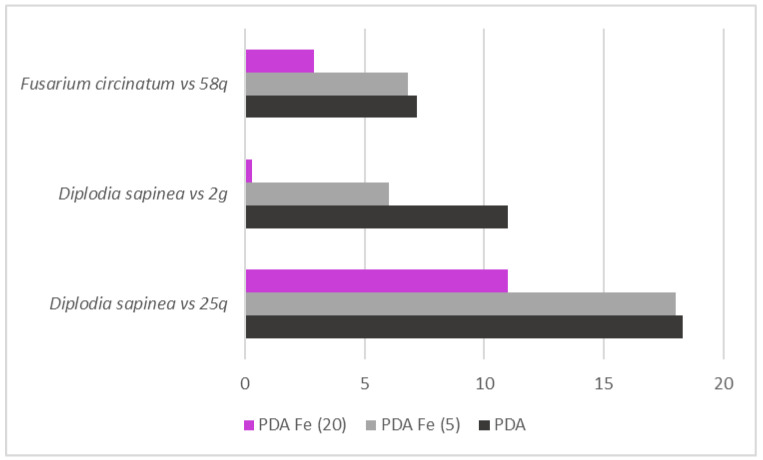
Effect of the iron concentration (5 = 5 µg/mL; 20 = 20 µg/mL) on the reduction in diameter of the pathogenic strains: *Diplodia sapinea,* in paired cultures with the 2g and 25q isolates, and *Fusarium circinatum,* in paired cultures with the 58q isolate.

Additionally, three of these selected yeasts (58q, 25q, and 2g) significantly increased their inhibitory capacity against pathogens in the presence of iron (F_6, 45_ = 1093.99, *p* < 0.001). Specifically, this inhibition was increased in 58q for *F. circinatum* and in 25q and 2g for *D. sapinea*, being greater at a higher concentration of this micronutrient (Figure 5). Moreover, 9g (*Metschnikowia pulcherrima*), 11g (*H. uvarum*), and 18g (*Metschnikowia fructicola*) isolates produced pigmented colonies, whose color intensity was augmented by the iron concentration in the presence and absence of pathogenic strains. The rest of the tested yeast did not show any visually detectable change in the pigment coloration of their colonies (Supplementary Figure S1f).

### 3.5. Phytopathogenicity Test on Higher Plants

We evaluated the potential pathogenicity of yeast isolates on higher plants using the Spirodela duckweed toxicity test and Phytotoxkit liquid samples. For these assays, we used the five yeast isolates selected in the previous section due to their inhibitory capacity against *F. circinatum* and *D. sapinea* in the in vitro test.

**Figure 6:**
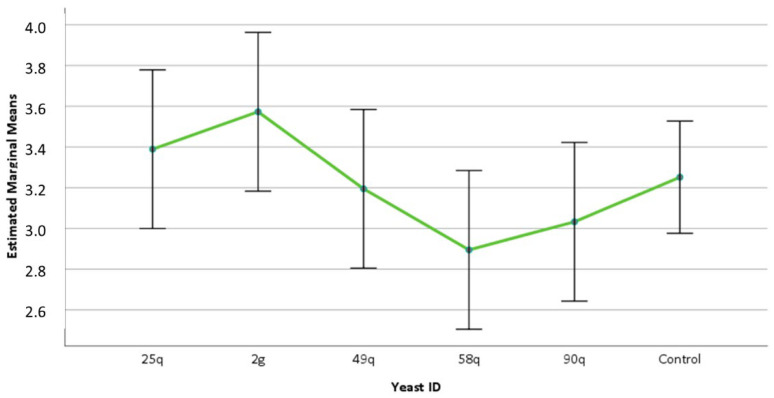
Mean growth rate of *Spirodela polyrhiza* turions after three days of treatment with the different yeast suspensions, compared to the control that was not treated with any yeast. Bars indicate error, 95% confidence intervals.

In the duckweed toxicity test, the impact of the yeast on the growth of the *Spirodela polyrhiza* activity was not statistically significant (F_5, 78_ = 0.712, *p* = 0.185), and no significant effect on turion growth attributable to the yeast suspensions or symptoms of diseases in any of them (chlorosis or rot) was detected (Figure 6 and Supplementary Figure S3).

**Figure 7:**
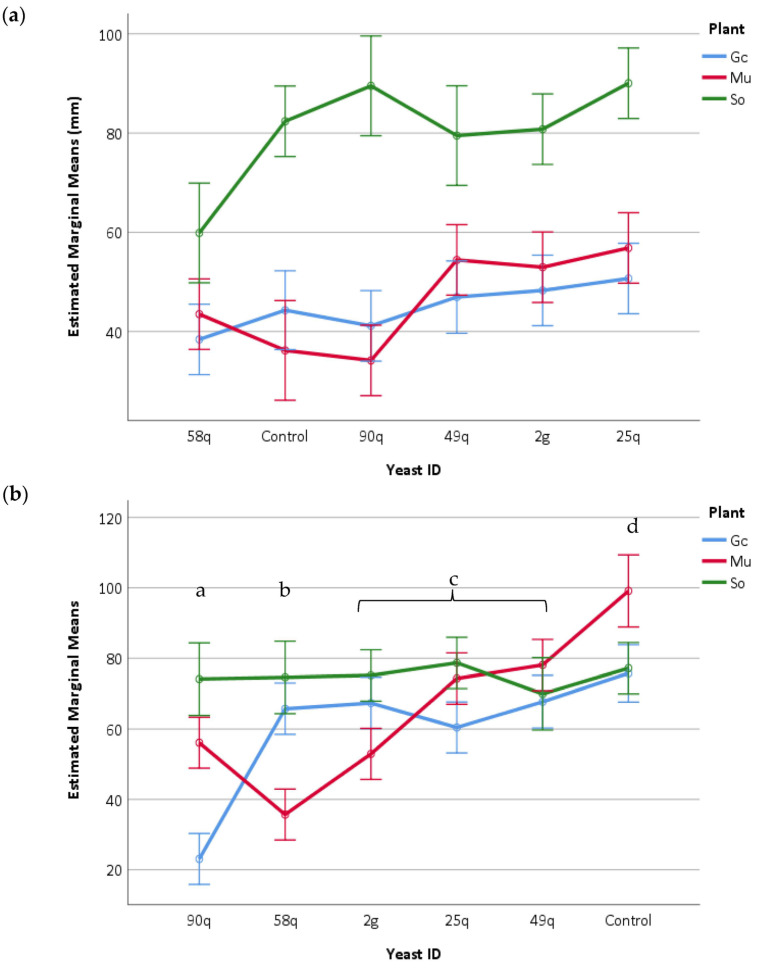
Length (mm) of the aerial part (**a**) and root system (**b**) of the plants (Gc = Garden cress, Mu = Mustard, and So = Sorghum) after having been treated with the different yeast suspensions, compared to the control treated with only water. Statistically significant differences (*p* < 0.05) in the effect of each yeast for the whole set of tested plants are indicated with different lowercase letters. Bars indicate error, 95% confidence intervals.

The Phytotoxkit test was performed in monocotyledonous (*Sorghum saccharatum*) and dicotyledonous (*Lepidium sativum* and *Sinapis alba*) plants. The application of the yeasts had no impact on the germination of the seeds of the tested monocotyledonous and dicotyledonous plants. None of the treated plants showed symptoms of decline such as chlorosis or rot (Supplementary Figure S4a). However, the plant species and the yeast isolates showed significant differences in the growth of the aerial (plant species: F_2, 297_ = 135.43, *p* < 0.001; yeast isolate: F_5, 297_ = 8.09, *p* < 0.001) and root parts (plant species: F_2, 297_ = 19.35, *p* < 0.05; yeast isolate: F_5, 297_ = 21.43, *p* < 0.05) of the plant. In addition, a statistically significant interaction between yeast isolates applied and plant species used in the test for the growth of the length of the aerial part of the plant (F_10, 297_ = 2.85, *p* < 0.05) and the length of the rooting system (F_10, 297_ = 14.27, *p* < 0.001) was detected (Figure 7). Even though the seeds responded to yeast treatment with a significant shortening of root length, as in the case of *S. alba* plants with 58q yeast application, an increase in secondary root development was observed, while plant vigor showed no signs of decline (Supplementary Figure S4b).

### 3.6. In Vivo Assays for Antagonistic Activity

All cuttings inoculated with *F. circinatum* and *D. sapinea* developed lesions. The most common symptom was an area of variable extension affected by necrosis around the inoculation point. In the case of *F. circinatum* inoculation, these lesions were accompanied by the characteristic abundant production of pitch. In addition, when the bark was removed, the tissue underneath was brownish. The control plants inoculated with water or yeast suspensions never produced any lesions other than the mechanical injury (< 1 mm) caused by the wounding procedure, evidencing the nonpathogenicity of yeasts in *P. radiata*.

The mean lesion lengths measured in the different experiments were initially compared by ANOVA, using all the variables jointly (plant clones, yeast isolates, and pathogenic fungi), and significant differences were found (*p* = < 0.0001).

**Figure 8:**
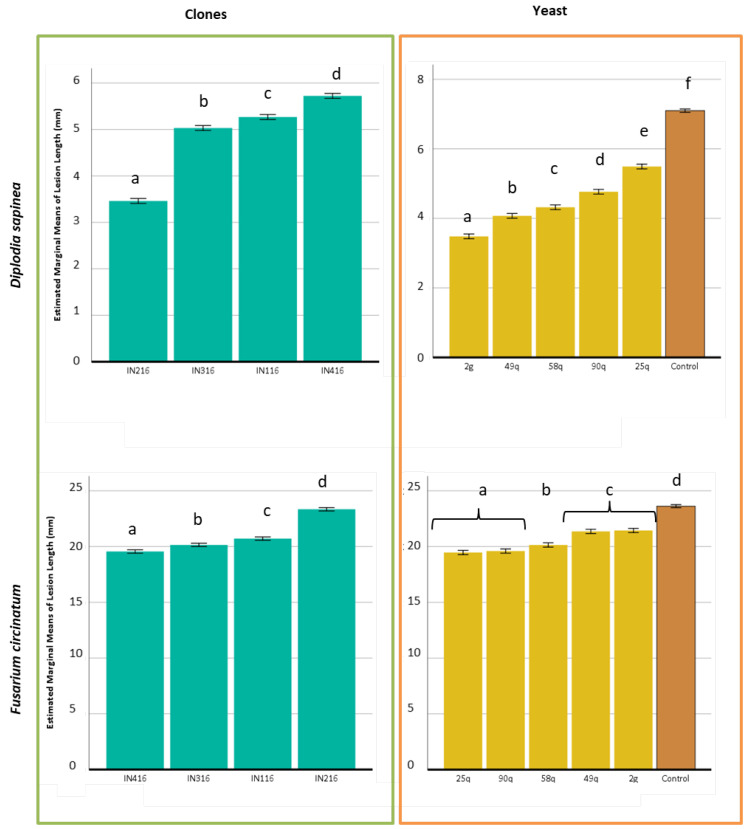
Length of the lesion caused by *Diplodia sapinea* (above) and *Fusarium circinatum* (below) in in vivo assays of clonal plants treated with a yeast suspension. Statistically significant differences of *p* < 0.05 among cuttings (left side) and yeast (right side). These differences are indicated with different lowercase letters. Bars indicate error, 95% confidence intervals.

*Fusarium circinatum* was the most virulent pathogen in terms of lesion length, with lesions twice and even three times longer than those obtained with *D. sapinea* (Figure 8). The clones responded significantly differently to the pathogen and yeast inoculations, showing significant differences in the lesion length. In the *F. circinatum* inoculations, IN216 plants were the most susceptible, followed by IN116, IN316, and IN416, which were the most tolerant. In contrast, the IN416 clone was the most susceptible, and IN216 was the most tolerant to *D. sapinea*. The application of yeast treatment also had a different response depending on the pathogen present. The 2g isolate (*T. delbrueckii*) showed the highest inhibitory activity for *D. sapinea* and the 25q, 90q (*S. paradoxus*) for *F. circinatum*, although the plant clone factor showed significant differences in the length of the lesions (*p* < 0.001) and there is an interaction between these two factors (Figure 8).

## 4. Discussion

The control of fungal diseases in nurseries and plantations has classically been based on treatments with chemical fungicides. However, the progressive loss of effectiveness against pathogens, the emergence of resistant strains, and the increasing level of residues in the environment have led the European Union to restrict or even ban the use of these compounds, leading researchers toward innovative and eco-friendly strategies to manage disease impact. In line with the guidance of UE Directive 128/2009, this study focused on the search for the natural antagonistic potential of 58 local wild yeasts isolated from different native oak species and grape samples of *Vitis vinifera* cv Tempranillo against *F. circinatum* and *D. sapinea*. In this research phase, we focused on the fungal pathogenic species that cause the main diseases in pine, pitch canker, and pine shot blight. *P. radiata* is one of the most widely planted tree species worldwide (>4 million ha) [43], the largest contributor to the forestry industry in northern Spain [44], and one of the pine species that is most susceptible to both organisms.

*Fusarium circinatum* and *D. sapinea* are organisms that are difficult to control due to their plant attack strategies and their ability to spread and survive for long periods under unfavorable conditions and even persist in saprophytic conditions [45,46]. In this sense, knowledge of the interaction mechanisms between pathogens and possible biocontrol agents is a key factor for the selection of such biocontrol agents and the development of biopesticides [47]. Yeasts are found in all environments and have been described as potent antagonists of several plant pathogens [48]. Therefore, it is important to understand the ways in which yeast acts against the pathogen to develop an appropriate formulation and application method [49]. Several mechanisms of action, such as the competition for nutrients and space, the secretion of enzymes, and the production of toxins and volatile organic compounds, are involved in the biocontrol processes of antagonistic yeasts [48]. In this study, the different modes of action that are present in yeast, such as ligninolytic and cellulolytic enzyme activity, the production of acetic acid and hydrogen sulfide, tolerance to biocides, and inhibitory activity against *F. circinatum* and *D. sapinea* in in vitro and in vivo assays, were examined.

The ligninolytic and cellulolytic enzyme activity of 58 antagonistic yeasts and their potential impact on representative higher plants were estimated to rule out their impact on the pathogenic host (*P. radiata*) and the surrounding vegetation and to evaluate their capacity as decomposers of woody biomass. This capacity is especially relevant to enhance the composting process and reduce the survival and propagation capacities of *D. sapinea* and *F. circinatum*. Both pathogens persist asymptomatically in living trees and in tree debris produced after pruning and plantation harvesting. The response of the studied yeasts to the degradation of lignin and cellulose was quite diverse and dependent on the strain. Although some strains, such as 90q or 6g, showed high activity in all the enzymes, a wide variety of enzyme profiles were obtained among the strains, supporting the idea of using mixtures of microorganisms rather than a single microorganism as a way to increase the effectiveness of the degradation process. This strategy could even include noncellulolytic and nonligninolytic microorganisms, such as hydrogen consumers or carbohydrate consumers, to improve the process [50,51,52]. These degradative activities of nonpathogenic yeasts that are capable of colonizing plant surfaces represent a competition strategy since they limit the amount of available nutrients, which reduces the spore germination rate of the pathogen and their invasion capacity of the host [53].

The application of biocides is one of the most effective methods to control microbial deterioration, and their tolerance is an interesting characteristic for microorganisms with biotechnological interest. In our study, four strains (51q, 96q, 103q, and 3g) showed the highest biocide tolerance, being slightly sensitive to only one of the tested biocides, while some strains (29q, 86q, 11g, and 20g) were sensitive to seven of the eight tested biocides. Most of them (86%) showed sensitivity to caspofungin, an antifungal drug and the first member of a new drug class called the echinocandins. It works by inhibiting β(1,3)-D-glucan of the fungal cell wall. This assay demonstrates the diverse capacity of these yeasts to tolerate the presence of these inhibitory substances. This must be taken into consideration when selecting one of these antagonistic yeasts as a biocontrol agent since its effectiveness can drop considerably, eventually disappearing or being replaced by other microorganisms with greater tolerance to a specific biocide.

The production of volatile organic compounds that can interact with pathogens, inhibiting or reducing their growth and infection, is considered another action mechanism of yeasts that is useful in biocontrol. In this study, yeast produced less acetic acid than hydrogen sulfide. Indeed, twenty-six percent of the yeast strains did not show detectable production of acetic acid, while practically all the strains produced hydrogen sulfide, and most in high concentrations. These molecules have additional advantages as biocontrol agents, such as the low concentration needed to be effective or the ability to spread and act on pathogens without physical contact [53]. Hydrogen sulfide activates resistance to diseases caused by pathogens and improves fruit resistance to postharvest stress [28]. On the other hand, acetic acid is an economical and environmentally friendly fungicide with a potentially wide scope of application, especially in organic agriculture, where conventional pesticides are prohibited [29].

The results obtained from the in vitro assay clearly revealed that five of the yeast isolates (25q, 49q, 58q, 90q, and 2g) can inhibit the growth of *F. circinatum* and *D. sapinea*. Moreover, this inhibition capacity increased in the presence of iron. Iron is an essential micronutrient whose availability is a limiting factor that promotes competition among microorganisms. Sipiczki [27] and Spadaro and Droby [49] reported this action on strains belonging to the *Metschnikowia* genus that were capable of stopping mold development in crop areas through an iron deficiency mechanism. To our knowledge, this is the first report evaluating the potential antagonistic capacity of yeasts isolated from *Quercus* species and *V. vinifera* against *D. sapinea* and *F. circinatum*. Additionally, in in vivo assays, significant differences in lesion length were observed depending on the pathogen infecting the plant but also depending on the yeast and plant clone. The application of selected yeasts in combination with the use of tolerant clones reinforces the protection of trees against pitch canker and pine shot blight, in consonance with the proposal of Prospero et al. [54]. Therefore, the treatments to be carried out will depend on the disease, since neither the yeasts nor the pine clones respond in the same way to inoculation with one pathogenic species or another. This leads us to have to consider the best option in the case of having to address situations in which both diseases are present at the same time, only one of them, or even the possibility of using a combination of these yeasts to increase the chances of success. It is important to highlight that these five strains presented good lignin and cellulose degradation activities, especially the 90q strain, which produced volatile organic compounds, especially hydrogen sulfide in the case of the 49q strain, and exhibited different tolerances to biocides, with 58q being the most tolerant. Therefore, combinations of these five strains may confer additive protection against pathogenic species.

## 5. Conclusions

This investigation on antagonism patterns in new yeast isolates can constitute a promising source of knowledge and experience to set strategies to prevent or reduce damage to harvested commodities. Specifically, 5 of the 58 yeast isolates studied, 2g (*T. delbrueckii*) isolated from *Vitis vinifera* and 25q, 49q, 90q (all *S. paradoxus* species), and 58q (*K. dobzhanskii*) from native oak ecosystems, have been selected as good candidates to be tested as substitutes for chemical fungicides. Biological control using yeast is a promising and environmentally friendly approach for disease management. However, to increase the chances of success of biological control in forest pathology, an integral approach is needed, combining the application of microorganisms with other sustainable management strategies (e.g., sanitation pruning and thinning, reducing plantation density, and enhancing the use of tolerant trees). In addition to controlling the preservation of the forest environment, it is imperative to implement efficient monitoring and use protective measures. Forest health should be constantly monitored to assess whether the beneficial and pathogenic microbial communities change. Moreover, further research is needed to select efficient yeast strains and determine the suitable timing of their application according to the type of plant material and environmental conditions.

## Figures and Tables

**Table 1:** Yeast isolates used in this study to evaluate their potential for biological control against *Fusarium circinatum* and *Diplodia sapinea.*

NCBI	ID Code	Location	Sample	Host	NCBI	ID Code	Location	Sample	Host
*Candida molendinolei*	30q	ULLIVARRI ARRAZUA	Acorn	*Qercus faginea*	*Pichia fermentans*	117q	ESKALMENDI	Bark	*Q. ilex*
*Candida subhashii*	102q	ESKALMENDI	Soil	*Q. robur*	*Pichia kudriavzevii*	142q	ESKALMENDI	Bark	*Q. ilex*
*Hanseniaspora uvarum*	11g	RIOJA	Grapes	*Vitis vinifera*		138q	ESKALMENDI	Acorn	*Q. robur*
*Hyphopichia pseudoburtonii*	6g	RIOJA	Grapes	*V. vinifera*		146q	ESKALMENDI	Soil	*Q. robur*
*Kluyveromyces dobzhanskii*	109q	ESKALMENDI	Acorn	*Q. ilex*		107q	ESKALMENDI	Bark	*Q. ilex*
	67q	LANDA	Leaves	*Q. robur*		7g	RIOJA	Grapes	*V. vinifera*
	45q	MURGIA	Acorn	*Q. robur*	*Pichia manshurica*	139q	ESKALMENDI	Soil	*Q. petraea*
	63q	LANDA	Acorn	*Q. robur*		136q	ESKALMENDI	Soil	*Q. robur*
	69q	LANDA	Acorn	*Q. robur*	*Saccharomyces cariocanus*	56q	IZARRA	Bark	*Q. robur*
	58q	IZARRA	Leaves	*Q. robur*	*Saccharomyces cerevisiae*	20g	RIOJA	Grapes	*V. vinifera*
*Kluyveromyces marxianus*	23q	ULLIVARRI ARRAZUA	Bark	*Q. faginea*	*Saccharomyces paradoxus*	19q	ESKALMENDI	Soil	*Q. ilex*
*Lachancea thermotolerans*	104q	ESKALMENDI	Leaves	*Q. faginea*		96q	ESKALMENDI	Soil	*Q. petraea*
	118q	ESKALMENDI	Acorn	*Q. robur*		46q	MURGIA	Soil	*Q. robur*
	51q	IZARRA	Soil	*Q. robur*		29q	ULLIVARRI ARRAZUA	Leaves	*Q. faginea*
	8g	RIOJA	Grapes	*V. vinifera*		21q	ULLIVARRI ARRAZUA	Soil	*Q. faginea*
	103q	ESKALMENDI	Acorn	*Q. ilex*		25q	ULLIVARRI ARRAZUA	Soil	*Q. faginea*
	163q	IZKI	Bark	*Q. pirenaica*		86q	SALINAS DE LENIZ	Bark	*Q. petraea*
	12g	RIOJA	Grapes	*V. vinifera*		90q	SALINAS DE LENIZ	Soil	*Q. petraea*
	13g	RIOJA	Grapes	*V. vinifera*		59q	LANDA	Acorn	*Q. robur*
	14g	RIOJA	Grapes	*V. vinifera*		43q	MURGIA	Bark	*Q. robur*
	15g	RIOJA	Grapes	*V. vinifera*		70q	LANDA	Bark	*Q. robur*
	16g	RIOJA	Grapes	*V. vinifera*		49q	IZARRA	Leaves	*Q. robur*
	19g	RIOJA	Grapes	*V. vinifera*		53q	IZARRA	Leaves	*Q. robur*
*Metschnikowia fructicola*	18g	RIOJA	Grapes	*V. vinifera*		68q	LANDA	Soil	*Q. robur*
*Metschnikowia pulcherrima*	9g	RIOJA	Grapes	*V. vinifera*	*Starmerella bacillaris*	4g	RIOJA	Grapes	*V. vinifera*
*Meyerozyma caribbica*	116q	ESKALMENDI	Bark	*Q. ilex*		10g	RIOJA	Grapes	*V. vinifera*
*Naganishia albida*	5g	RIOJA	Grapes	*V. vinifera*	*Torulaspora delbrueckii*	22q	ULLIVARRI ARRAZUA	Bark	*Q. faginea*
*Ogataea dorogensis*	1q	ARKAUTE	Leaves	*Q. robur*		2g	RIOJA	Grapes	*V. vinifera*
*Wickerhamomyces anomalus*	1g	RIOJA	Grapes	*V. vinifera*		3g	RIOJA	Grapes	*V. vinifera*

## Data Availability

Data is contained within the article or supplementary material.

## Supplementary Materials

The following supporting information can be downloaded at https://www.mdpi.com/article/10.3390/jof9080840/s1, Figure S1: (a) Ligninolytic enzymatic activity of the yeast showed by the decolored areas growing in Remazol brilliant blue and Aniline blue agar media. (b) Cellulolytic enzymatic activity of the yeast showed by the decolored areas growing in CMC-Na agar medium. (c) Inhibition of yeast growth by the antimicrobial agents included in the multidisc system, yeast Multidisc 95280. (d) Yeast growing in Biggy medium showing the qualitative among of H_2_S production delimited by the colors. (e) Determination of acetic acid production by the presence of a halo around the colony growing in CaCO_3_ media. (f) Effect of iron concentration on the inhibitory activity of the yeasts against the pathogen *Diplodia Sapinea* in PDA plates (left), PDA with 5 µg/mL of FeCl_3_ (center) and PDA with 20 µg/mL of FeCl_3_ (right). Figure S2: (a) *F. circinatum* (left) and *D. sapinea* (right) pure cultures growing in PDA Petri Plates. (b) Different responses of both pathogens to the pairing with yeast isolates. *D. sapinea* (above) and *F. circinatum* (below). Figure S3: (a) Incubation of the Petri Dish with Steinberg medium for 72 h at 25 °C under continuous top illumination of 6000 lux to germinate the *Spirodela polyrhiza* turions before exposure them to the tested yeast suspensions. (b) Germinated turions in multiwell test plates at start of the tests. (c) Grown turions at the end of the experiment. Figure S4: Essay of the potential pathogenicity of wild yeast application on plant. (a) Germination of the three species of seeds, without any treatment (control), from the left to the right, in the monocotyledonous *Sorghum saccharatum*, and the dicotyledonous *Lepidium sativum* and *Sinapis alba*. (b) Differences in the secondary and main rooting system of *Sinapis alba* plants with application of 58q yeast and the control without yeast suspension. Table S1: Results of ligninolytic and cellulolytic activity, biocide tests and production of volatile organic compounds (hydrogen sulfide and acetic acid) of the 58 yeast isolates.
