# Supplementary material for: The Potential of Wild Yeasts as Promising Biocontrol Agents against Pine Canker Diseases
Source: J Fungi (Basel). 2023 Aug 11;9(8):840. doi: 10.3390/jof9080840 (PMC10455916; doi:10.3390/jof9080840)
Supplement: Supplementary file 1 [file jof-09-00840-s001.zip › jof-2529873-supplementary.pdf]

Supplementary Material

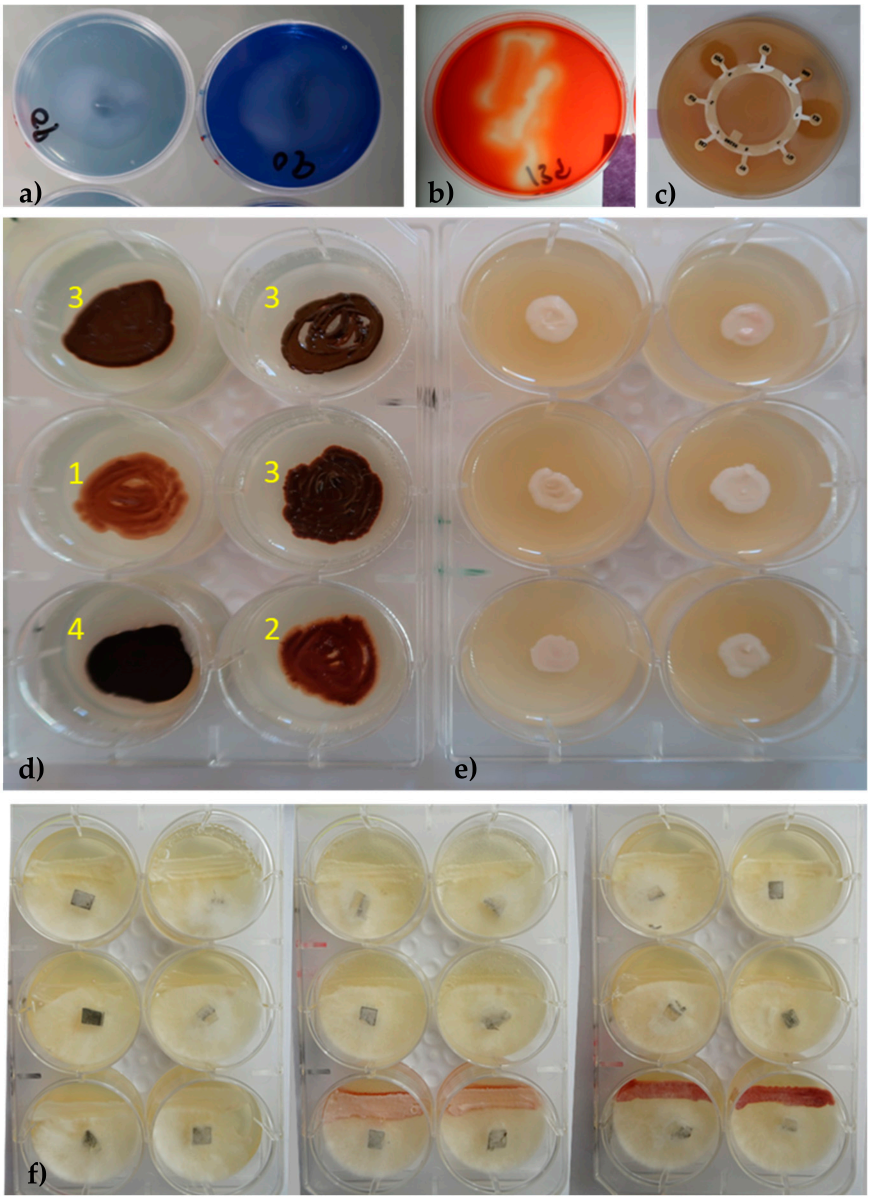

Figure S1. a) Ligninolytic enzimatic activity of the yeast showed by the decoloured areas growing in Remazol brilliant blue and Aniline blue agar media. b) Cellulolytic enzimatic activity of the yeast showed by the decoloured areas growing in CMC-Na agar medium. c) Inhibition of yeast growth by the antimicrobial agents included in the multidisc system, yeast Multidisc 95280. d) Yeast growing in Biggy medium showing the qualitative among of H_2S production delimited by the colors. e) Determination of acetic acid production by the presence of a halo around the colony growing in $CaCO_3$ media. f) Effect of iron concentration on the inhibitory activity of the yeasts against the pathogen *Diplodia Sapinea* in PDA plates (left), PDA with 5 µg/ml of $FeCl_3$ (center) and PDA with 20 µg/ml of $FeCl_3$ (right).

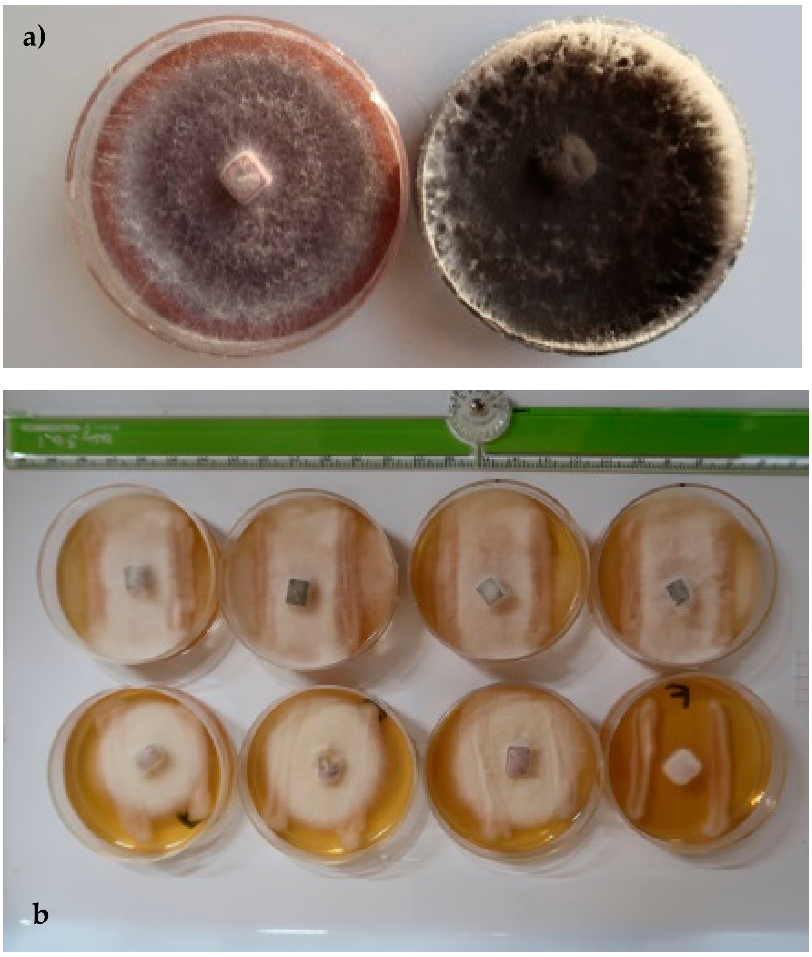

Figure S2. a) *F. circinatum* (left) and *D. sapinea* (right) pure cultures growing in PDA Petri Plates. b) Different responses of both pathogens to the pairing with yeast isolates. *D. sapinea* (above) and *F. circinatum* (below).

a)

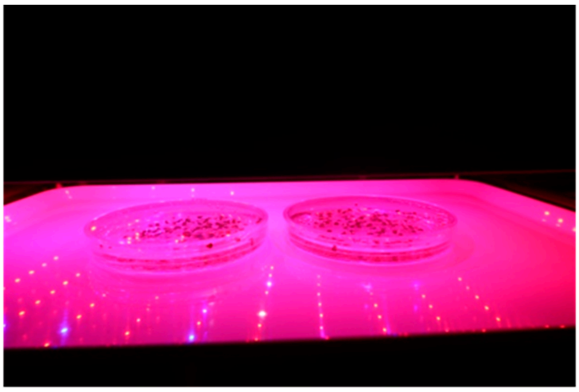

b) **c)**

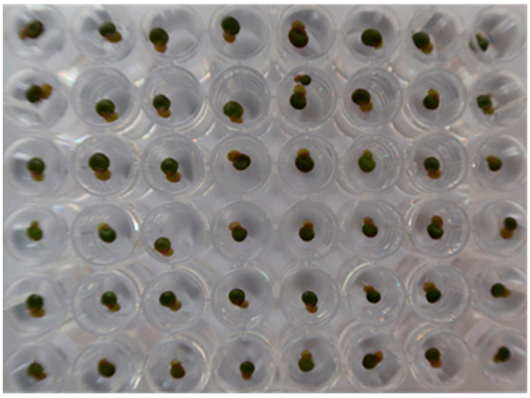

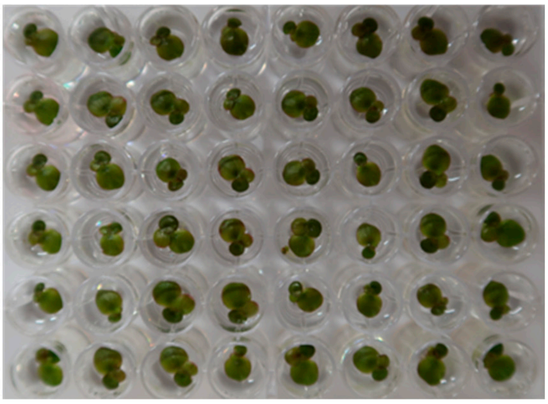

Figure S3. a) Incubation of the Petri Dish with Steinberg medium for 72h at 25°C under continuous top illumination of 6000 lux to germinate the *Spirodela polyrhiza* turions before exposure them to the tested yeast suspensions. b) Germinated turions in multiwell test plates at start of the tests c) Grown turions at the end of the experiment.

a)

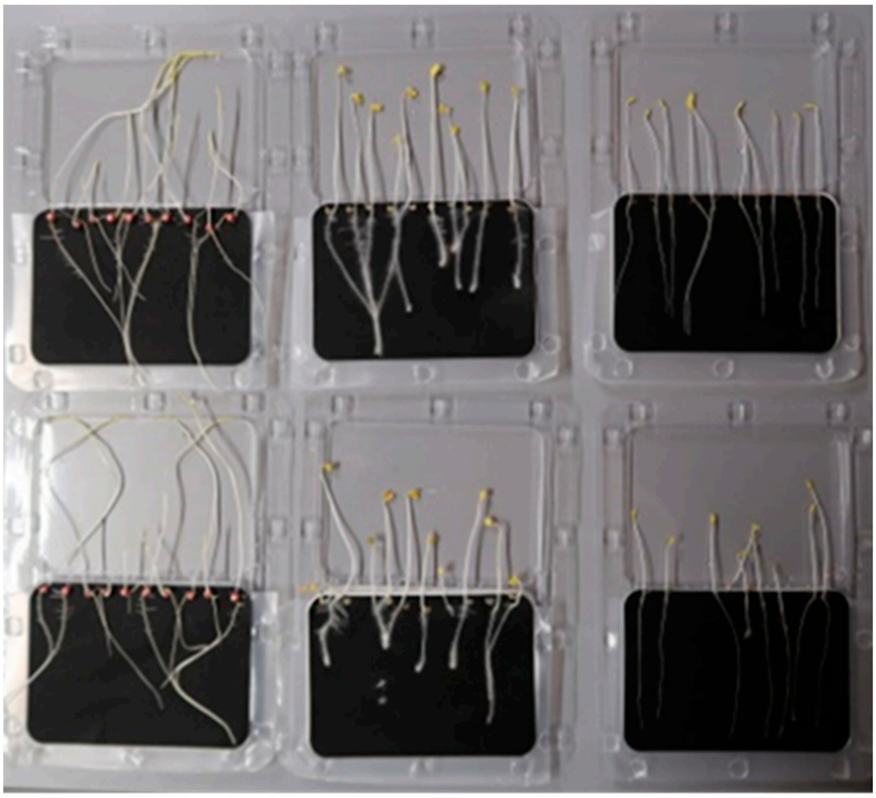

b)

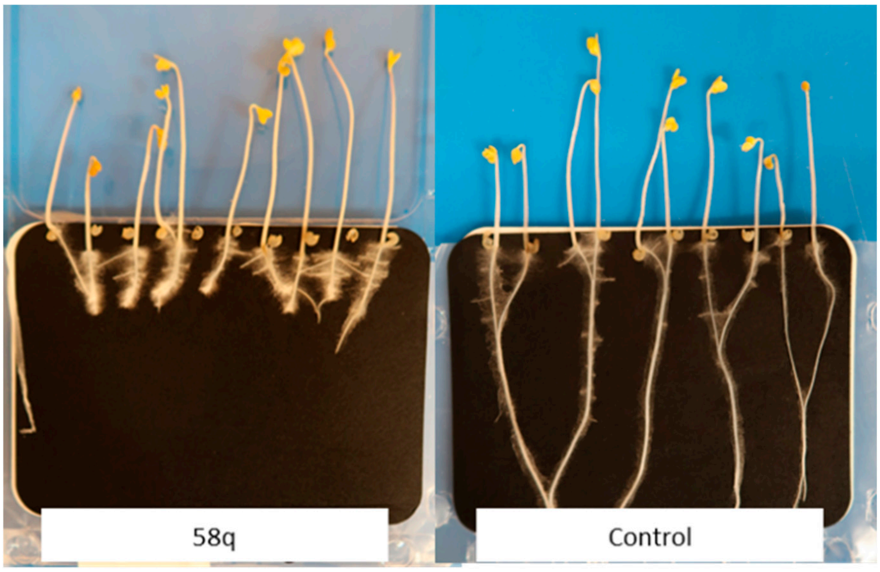

Figure S4. Essay of the potential pathogenicity of wild yeast application on plant. a) Germination of the three species of seeds, without any treatment (control), from the left to the right, in the monocotyledonous *Sorghum saccharatum*, and the dicotyledonous *Lepidium sativum* and *Sinapis alba*. b) Differences in the secondary and main rooting system of *Sinapis alba* plants with application of 58q yeast and the control without yeast suspension.

Table S1. Results of ligninolytic and cellulolytic activity, biocide tests and production of volatile organic compounds (hydrogen sulphde and acetic acid) of the 58 yeast isolates.

ID	Lignin II (mm)	Lignin III (mm)	Cellulose (mm)	CAS (5 µg)	FLU (25 µg)	POS (5 µg)	VO (1 µg)	AMB (20 µg)	KCA (10 µg)	AFY (1 µg)	NY (100 µg)	H2S released	Acetic acid production
**1q**	0	6.47	3.99	4	0	3	3	0	4	0	0	3	1
**19q**	3.98	3.75	3.92	3	0	1	0	0	4	0	2	3	2
**21q**	8	7.2	3.51	4	0	0	0	0	4	0	2	4	2
**22q**	5.09	9.18	2.09	1	0	0	0	0	0	0	1	1	1
**23q**	3.19	4.76	3.79	0	0	2	0	0	0	0	0	2	1
**25q**	6.92	3.05	6.12	3	0	3	1	0	4	4	2	3	1
**29q**	6.48	2.78	3.48	4	0	2	1	1	4	1	3	3	2
**30q**	3.17	4.72	6.23	4	0	4	0	0	4	0	0	2	2
**43q**	4.52	5	2.07	3	0	0	0	0	0	0	0	2	2
**45q**	4.37	4.51	5.39	2	0	0	0	0	0	0	1	2	0
**46q**	4.35	9.88	2.78	4	0	2	0	0	0	0	1	2	0
**49q**	6.52	2.63	2.80	3	1	3	3	0	4	0	2	4	0
**51q**	9.67	2.29	3.99	1	0	0	0	0	0	0	0	3	2
**53q**	6.33	1.77	5.28	4	0	2	0	0	1	1	2	4	0
**56q**	3.5	3.38	3.61	2	0	0	0	0	0	0	2	2	0
**58q**	4.02	4.22	5.19	3	0	0	0	0	0	0	0	2	2
**59q**	4.81	3.45	3.19	3	0	0	0	0	0	0	1	2	1
**67q**	3.85	5.98	5.62	3	0	0	0	0	0	0	0	4	1
**68q**	4.79	4.32	2.88	4	0	0	2	0	0	0	2	4	2
**69q**	6.91	7.15	6.09	4	0	0	0	0	0	0	1	4	1
**70q**	5.42	6.48	3.67	4	1	1	0	0	4	0	1	2	1
**86q**	4.06	2.79	2.49	4	2	4	1	2	4	0	3	4	0

**90q**	9.78	8.92	4.48	4	0	0	0	0	4	1	2	2	2
**96q**	3.68	5.93	7.69	0	0	0	0	0	0	0	1	1	1
**102q**	5.18	4.71	5.31	2	0	3	2	0	4	0	1	1	1
**103q**	6.7	7.68	4.16	0	0	0	0	0	0	0	1	4	0
**104q**	0	0	3.18	3	3	2	0	3	4	0	2	2	1
**107q**	0	0	4.95	2	0	0	0	0	0	0	0	4	1
**109q**	6.39	3.99	5.37	4	0	0	0	0	0	0	2	2	2
**116q**	5.58	3.32	7.19	1	0	0	1	0	1	0	0	3	0
**117q**	3.72	1.35	2.39	0	0	2	3	0	4	0	0	4	2
**118q**	7.98	1.19	4.44	2	0	4	0	1	4	0	1	3	0
**136q**	0	0	2.51	4	0	4	0	0	0	0	0	3	1
**138q**	5.81	3.61	3.55	4	1	3	0	0	3	0	0	4	1
**139q**	0	0	14.39	0	0	3	0	0	4	1	0	4	2
**142q**	4.99	5.13	2.61	4	1	4	0	2	0	1	3	4	1
**146q**	0	1.99	3.13	4	2	3	0	0	4	0	0	4	1
**163q**	4.62	4.21	4.87	3	0	1	0	2	2	0	0	4	1
**10g**	2.93	1.22	2.69	1	0	3	0	0	4	3	2	0	0
**11g**	4.29	1.49	3.37	4	1	4	4	0	4	2	1	2	0
**12g**	3.18	5.8	3.06	3	4	3	3	1	4	0	0	3	2
**13g**	5.73	0	3.45	3	2	1	3	0	0	0	2	1	2
**14g**	0.61	1.42	3.38	2	3	2	3	0	4	0	1	3	0
**15g**	3.16	4.55	4.19	4	1	2	0	1	4	0	0	4	2
**16g**	7.63	3.29	3.81	4	0	3	0	0	4	0	3	3	1
**17g**	7.38	2	4.43	4	0	2	0	0	4	0	3	4	2
**18g**	2.22	1.46	4.69	0	0	4	1	0	0	0	1	4	2
**1g**	3.6	5.55	6.54	4	0	0	2	1	1	0	0	2	0
**20g**	6.35	7.96	3.48	4	4	0	1	2	3	1	1	1	2

**2g**	5.4	3.21	4.11	4	0	0	0	0	0	4	3	3	2
**3g**	2.31	6.12	5.19	0	0	0	0	0	0	0	1	3	0
**4g**	4.28	5.04	0.19	2	0	0	4	1	4	0	3	4	2
**5g**	8.71	4.11	3.49	3	0	0	2	2	2	4	2	2	1
**6g**	8.32	6	5.80	4	0	4	4	2	4	4	0	2	2
**7g**	6.91	3.94	2.35	4	0	3	2	0	4	0	0	4	1
**8g**	1.68	2.18	2.22	4	0	2	1	1	0	0	4	3	0
**9g**	1.77	1.27	3.51	0	1	4	4	0	4	0	2	2	1

Diameter of the halos indicated the effectiveness of the biocidal agent is classified into 4 categories depending on the size: Code 1) 2 mm; 2) >2-4 mm; 3) >4-6mm; 4) >6mm. The qualitative amount of H_2S production was determined by the colour of the colonies, ranging from white (no release) to black (high release). The codification used was: 0) white (non-detected), 1) light brown, 2) brown, 3) dark brown and 4) black. In the case of acetic acid, a halo around colonies growing on $CaCO_3$ agar medium means that the yeast isolates produce acetic acid. The isolates were classified according to the diameter of the halo: 0) no acetic acid detected 1) ≤ 1 mm 2) >1mm and ≤ 2mm.
